# ASCVac-1, a Multi-Peptide Chimeric Vaccine, Protects Mice Against *Ascaris suum* Infection

**DOI:** 10.3389/fimmu.2021.788185

**Published:** 2021-12-21

**Authors:** Ana Clara Gazzinelli-Guimarães, Denise Silva Nogueira, Chiara Cássia Oliveira Amorim, Fabrício Marcus Silva Oliveira, Anderson Coqueiro-Dos-Santos, Samuel Alexandre Pimenta Carvalho, Lucas Kraemer, Fernando Sérgio Barbosa, Vanessa Gomes Fraga, Flaviane Vieira Santos, Joseane Camilla de Castro, Remo Castro Russo, Milena Apetito Akamatsu, Paulo Lee Ho, Maria Elena Bottazzi, Peter J. Hotez, Bin Zhan, Daniella Castanheira Bartholomeu, Lilian Lacerda Bueno, Ricardo Toshio Fujiwara

**Affiliations:** ^1^ Biological Sciences Institute, Federal University of Minas Gerais, Belo Horizonte, Brazil; ^2^ BioIndustrial Division, Butantan Institute, Sao Paulo Secretary of Health, São Paulo, Brazil; ^3^ Texas Children’s Hospital Center for Vaccine Development, Department of Pediatric Tropical Medicine, National School of Tropical Medicine, Baylor College of Medicine, Houston, TX, United States

**Keywords:** epitopes, vaccine, chimera, vaccinology, ascariasis

## Abstract

Control of human ascariasis, the most prevalent neglected tropical disease globally affecting 450 million people, mostly relies on mass drug administration of anthelmintics. However, chemotherapy alone is not efficient due to the high re-infection rate for people who live in the endemic area. The development of a vaccine that reduces the intensity of infection and maintains lower morbidity should be the primary target for infection control. Previously, our group demonstrated that immunization with crude *Ascaris* antigens in mice induced an IgG-mediated protective response with significant worm reduction. Here, we aimed to develop a multipeptide chimera vaccine based on conserved B-cell epitopes predicted from 17 common helminth proteomes using a bioinformatics algorithm. More than 480 B-cell epitopes were identified that are conserved in all 17 helminths. The *Ascaris*-specific epitopes were selected based on their reactivity to the pooled sera of mice immunized with *Ascaris* crude antigens or infected three times with *A. suum* infective eggs. The top 35 peptides with the strongest reactivity to *Ascaris* immune serum were selected to construct a chimeric antigen connected in sequence based on conformation. This chimera, called ASCVac-1, was produced as a soluble recombinant protein in an *Escherichia coli* expression system and, formulated with MPLA, was used to immunize mice. Mice immunized with ASCVac-1/MPLA showed around 50% reduced larvae production in the lungs after being challenged with *A. suum* infective eggs, along with significantly reduced inflammation and lung tissue/function damage. The reduced parasite count and pathology in infected lungs were associated with strong Th2 immune responses characterized by the high titers of antigen-specific IgG and its subclasses (IgG1, IgG2a, and IgG3) in the sera and significantly increased IL-4, IL-5, IL-13 levels in lung tissues. The reduced IL-33 titers and stimulated eosinophils were also observed in lung tissues and may also contribute to the ASCVac-1-induced protection. Taken together, the preclinical trial with ASCVac-1 chimera in a mouse model demonstrated its significant vaccine efficacy associated with strong IgG-based Th2 responses, without IgE induction, thus reducing the risk of an allergic response. All results suggest that the multiepitope-based ASCVac-1 chimera is a promising vaccine candidate against *Ascaris* sp. infections.

## Introduction

Human ascariasis is one of the most common intestinal parasitic diseases in developing countries. Recent studies estimate that approximately 450 million people are infected worldwide (1), and this high prevalence is strongly associated with poverty and precarious health conditions, mainly in tropical and subtropical areas of sub‐Saharan Africa, Southeast Asia, and South America (2–4). According to the DALY index, which takes into account the sum of years of life lost by premature death and the number of years lived with disability, in 2019, ascariasis contributed to a loss of 0.754 million years worldwide (5).

There is still no vaccine available for *Ascaris*, and therefore mass drug administration is currently the main form of infection control in humans and pigs, one of the most important animal hosts. However, high rates of re-infection are commonly observed, even after successful treatment. To improve the quality of life, reducing morbidity in endemic areas, the development of a vaccine that reduces the intensity of infection or provides long-term protection should be the primary goal of parasite control. Thus, the use of new technologies, such as the production of recombinant proteins, the construction of synthetic genes, and multivalent, chimeric vaccines consisting of several antigens, will facilitate the characterization of proteins and allow the production of large amounts and varieties of antigens in the laboratory and beyond.

Through a literature search, we identified several protective antigens from *A. suum*, a parasite that infects both humans and pigs and shows 99% genetic similarity with the human *A. lumbricoides* parasite (6). These antigens include As14 (7), As16 (8, 9), As24 (10), As37 (11, 12), enolase-1 (13), AsPPase (14), and BOT (15), which have been expressed as recombinant proteins in different expression systems and used in preclinical trials. As described in a recent study (16), immunization with crude extract of adult *A. suum* worms (ExAD), crude extract of adult worm cuticles (CUT), and crude extract of infective larvae (L3) (ExL3) provoked a B-cell specific response in BALB/c mice, leading to high levels of IgG and a 60% worm reduction (16). Thus, it is of paramount importance to gain a better understanding of how *Ascaris* antigens induce host protective immunity and the mechanisms underlying said protection.

Through a comprehensive bioinformatics screening system, we identified and characterized 35 immunogenic B-cell epitopes. These B-cell epitopes were designed and combined in a chimera called ASCVac-1 based on their optimal alignment, based on predicted conformation. The construct was expressed as a recombinant protein for testing in BALB/c mice against challenge with *A. suum* infective eggs. The objective was to evaluate vaccine efficacy as well as to characterize the immunological, physiological, and immunopathological responses after immunization.

Our main findings were that immunization with ASCVac-1, adjuvanted with MPLA, induced a Th2 polarized response in the immunized mice, with a significant increase of IL-4, IL-5, and IL-13 levels and downregulation of IL-33. Meanwhile, ASCVac-1-immunized mice showed reduced neutrophils, but increased lymphocyte influx into the airways with reduced lower tissue damage, and consequently attenuated lung dysfunction. Taken together, this preclinical trial with ASCVac-1 chimera demonstrated significant protection against *Ascaris* infection associated with a Th2 dominant immune response and reduced pulmonary inflammation caused by *A. suum* larval migration, providing a potentially effective vaccine for the control of ascariasis.

## Materials and Methods

### Selection of B-Cell Epitopes by the Bioinformatic Pipelines

**Figure 1:**
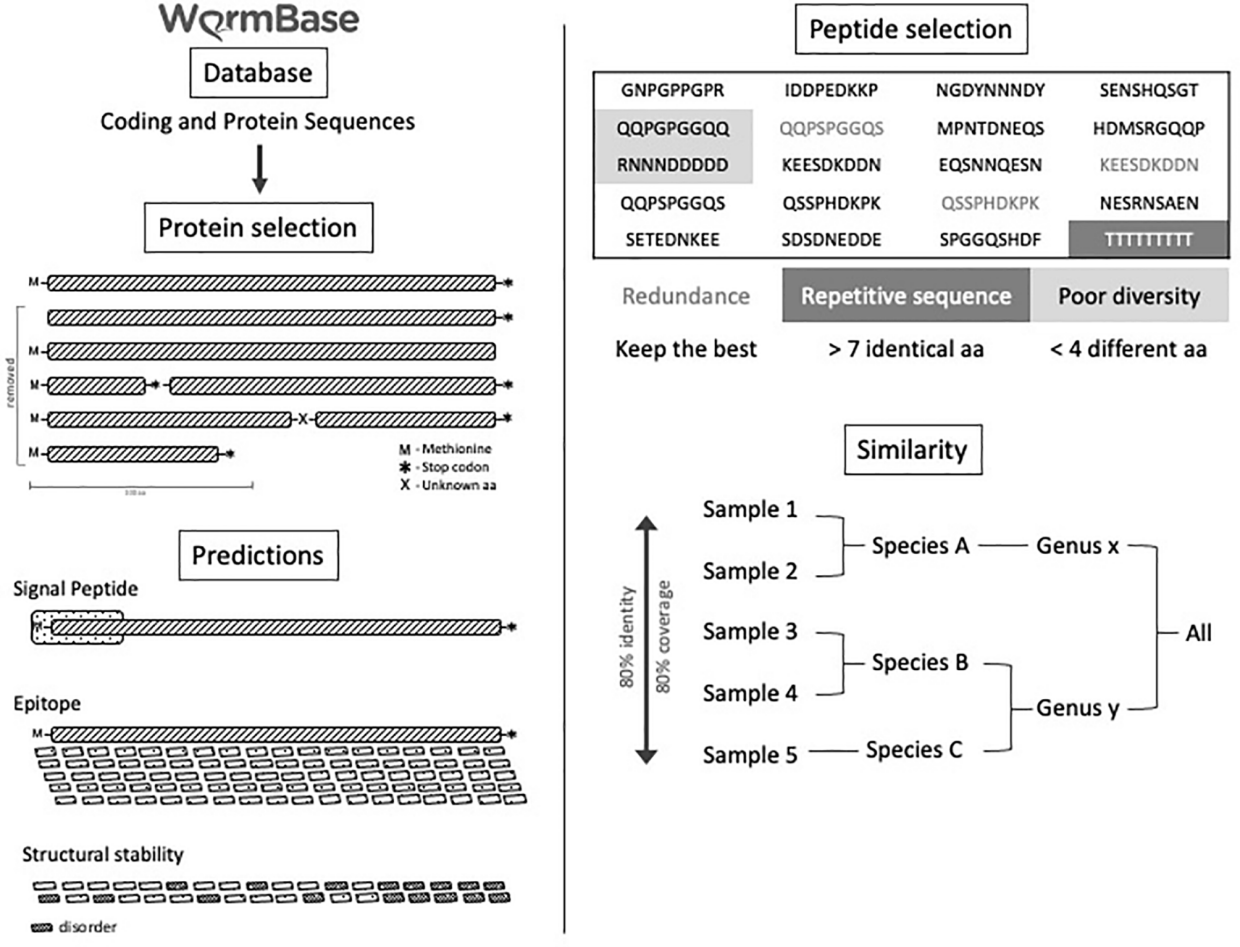
Experimental B-cell prediction through a bioinformatics pipeline. Experimental design of B-cell predicted-epitopes selected by data mining in a bioinformatics pipeline.

All proteome information was obtained from the WormBase ParaSite database WBPS10 version. The proteome information includes 17 species of helminths from the genera *Ancylostoma* (*Ancylostoma caninum*, *Ancylostoma ceylanicum*, and *Ancylostoma duodenale*), *Ascaris* (*Ascaris suum* and *Ascaris lumbricoides*), *Schistosoma* (*Schistosoma mansoni*), *Strongyloides* (*Strongyloides papillosus, Strongyloides ratti, Strongyloides stercoralis*, and *Strongyloides venezuelensis*), *Toxocara* (*Toxocara canis*), *Trichuris* (*Trichuris trichiura*), and *Wuchereria* (*Wuchereria bancrofti*). The encoded proteins from each genome were filtered by excluding those lacking an initial codon for methionine or a final stop-codon, or a complete reading frame, or those with sequences smaller than 100 amino acids. Proteins without a signal peptide predicted by SignalP 3.0, Markov’s hidden model, and the neural network’s method were also excluded. B-cell epitopes were predicted from the remaining proteins from each worm genome using Bepipred and IUpred predictors. For each helminth, 600 predicted B-cell epitopes with the highest scores were selected. Identical epitopes present in the same helminth were removed. Only B cell-predicted epitopes conserved among all 17 helminths were finally selected. After these analyses, the samples of the same genus were analyzed, and epitopes were recovered with a coverage of 85% and identity of 100% as evaluated by BlastP. In all stages, the highest scores were obtained for each of the epitopes. For duplicated epitopes, the highest prediction scores were considered. Some epitopes were discarded, taking into account the size, being accepted only 9 amino acids (maximum size generated by the predictor), and with the presence of greater than 7 of the same amino acid residues in the sequence. Finally, a table was generated with all epitopes by gender and with their respective scores. Finally, 480 B-cell predicted peptides that were conserved among the 17 helminth parasites were selected and screened by immunoblot for immunogenicity using helminth-reactive sera (**Figure 1**).

### Immunoblot Technique With Selected Peptides

The 480 B-cell predicted peptides (30 nmole each) conserved among 17 helminth parasites were each synthesized by the “Spot-Synthesis” method on a cellulose membrane with a 4.5 mm diameter. Peptides were tested for their specific reactivities to the sera from mice infected with *Ascaris* infective eggs three times (hyperimmune sera) or immunized with *Ascaris* adult worm crude antigens. Sera from normal mice were used as a negative control. Briefly, the membrane dotted with individual synthesized peptides was blocked with blocking solution (1xPBS-BSA 3%, Sucrose 4%) for 12–16 h at room temperature under agitation. On the next day, the membrane was washed three times using washing solution (1xPBS-0.1% Tween 20) for 10 min each wash, then incubated with *Ascaris*-immune-sera from mice (infected three times or immunized with adult crude antigens) or normal mouse control sera diluted 1:1,000 in 50 ml of washing solution. The membrane was washed three times and then incubated with anti-mouse IgG/HRP secondary antibody at 1:10,000 dilution in 50 ml of washing solution for 1 h at room temperature. After being washed three times, the membrane was visualized by Luminata revealing solution (Millipore/Merck, USA) and automatically exposed using the ImageQuant LAS4000 instrument. Our focus was to locate those peptides recognized strongly by *A. suum* immunized mouse sera or *Ascaris* crude antigen immunized mouse sera compared to the sera of negative control mice.

### Selection of Peptides Recognized by Mouse Immune Sera

The recognition intensity of individual peptides by the *Ascaris*-specific sera was quantitatively measured by densitometry scanning analysis using the Software Image J- Protein Array Analyzer. Through the densitometry analysis, we were able to characterize the reactivity of each peptide to *Ascaris*-immune sera compared to the normal mouse sera control. The *Ascaris*-immune sera–recognized positive peptides were selected based on the following selection criteria (1): Cutoff value (four spots with the lowest densitometry value were selected to calculate the mean as the cutoff value) (2); exclusion of negative peptides (cutoff value + 2× standard deviation, SD) (3); exclusion of barely positive peptides (cutoff value + 3× SD) (4); the densitometry value ≥ cutoff value 3× SD were selected as positive peptides. Based on these criteria, 35 peptides with the highest reaction to *Ascaris*-immune sera were selected as *Ascaris*-reactive peptides for the following experiments.

### Chimeric Protein Construction

Thirty-five positive peptides with the highest reaction to the *Ascaris*-immune sera were selected for constructing a chimeric vaccine candidate against *Ascaris* infection. The sequence of the 35 peptides was arranged based on their best conformation predicted by Immunorank Bioinformatics Analysis using 10,000 possible sequence arrangements. The best sequence arrangement of 35 peptides was further chosen based on its immunogenicity index. The final 35 selected linear B-cell epitopes were connected by lysine (KK) linkers as flexible connectors to generate the peptide-based chimera vaccine: ASCVac-1. It has been reported that KK linkers preserve independent immune responses when they are inserted between epitopes (17). The DNA coding for the chimera was codon-optimized based on *E. coli* codon usage using Graphical Codon Usage Analyser, with a TAA stop codon added to the end of the sequence, was synthesized by GenScript and cloned into the *E. coli* expression vector pET28a-TEV using NdeI and HindIII restriction sites.

### Protein Expression and Purification

The chimeric protein was subcloned into the bacterial expression vector pET28a-TEV. The recombinant plasmid was transformed into *E. coli* BL21 by electroporation and selected on LB agar plate containing 50 µg/ml kanamycin. The transformants with the correct insert were confirmed by PCR with vector flanking primers T7 and T7t. The small-scale expression of recombinant proteins in each transformant was induced with 1 mM IPTG. One of the transformants with the highest expression yield was used for large-scale expression. Briefly, 3 L of 2XYT medium containing 50 µg/ml kanamycin was inoculated with 150 ml overnight culture of the selected clone. When the culture reached an OD of 0.6, IPTG was added to a final concentration of 1 mM to induce the expression of the recombinant protein at 37°C with 180 rpm shaking overnight. Subsequently, the culture was centrifuged at 2,000 × g for 30 min at 4°C. The pellet was suspended in PBS buffer containing 30 mM imidazole and 10 mg/ml lysozyme. The complete lysis of the bacteria was achieved using an EmulsiFlex-C3 homogenizer. The expressed recombinant protein with a His-Tag at its N-terminus was purified by immobilized metal affinity chromatography using the AKTA System. Western blot with anti-His antibodies was performed to confirm the expressed chimeric protein containing His-tag. The recognition of the expressed chimeric protein by serum from mice infected with *A. suum* was also confirmed by ELISA.

### Parasites

*A. suum* adult worms were collected from the intestines of infected pigs from a slaughterhouse located in the city of Belo Horizonte, Minas Gerais, Brazil. Adult worms were kept in 0.4 M NaCl and 10 mM NaPO_4_, pH7.4, and taken to the Laboratory of Immunology and Genomics of Parasites of the Federal University of Minas Gerais to be processed. The eggs were isolated from the uteri of female adult worms by mechanical maceration, filtrated through a 100 μm nylon strainer, and placed in 50 ml of 0.2 M sulfuric acid in a culture flask at a concentration of 25 eggs/μl. The flask was stored in a BOD incubator at 26°C. On the 150^th^ day of culture, the eggs were fully embryonated and had reached the peak of larvae infectivity and were ready for experimental infections (18).

### Vaccination Protocol and *A. suum* Infection

**Figure 2:**
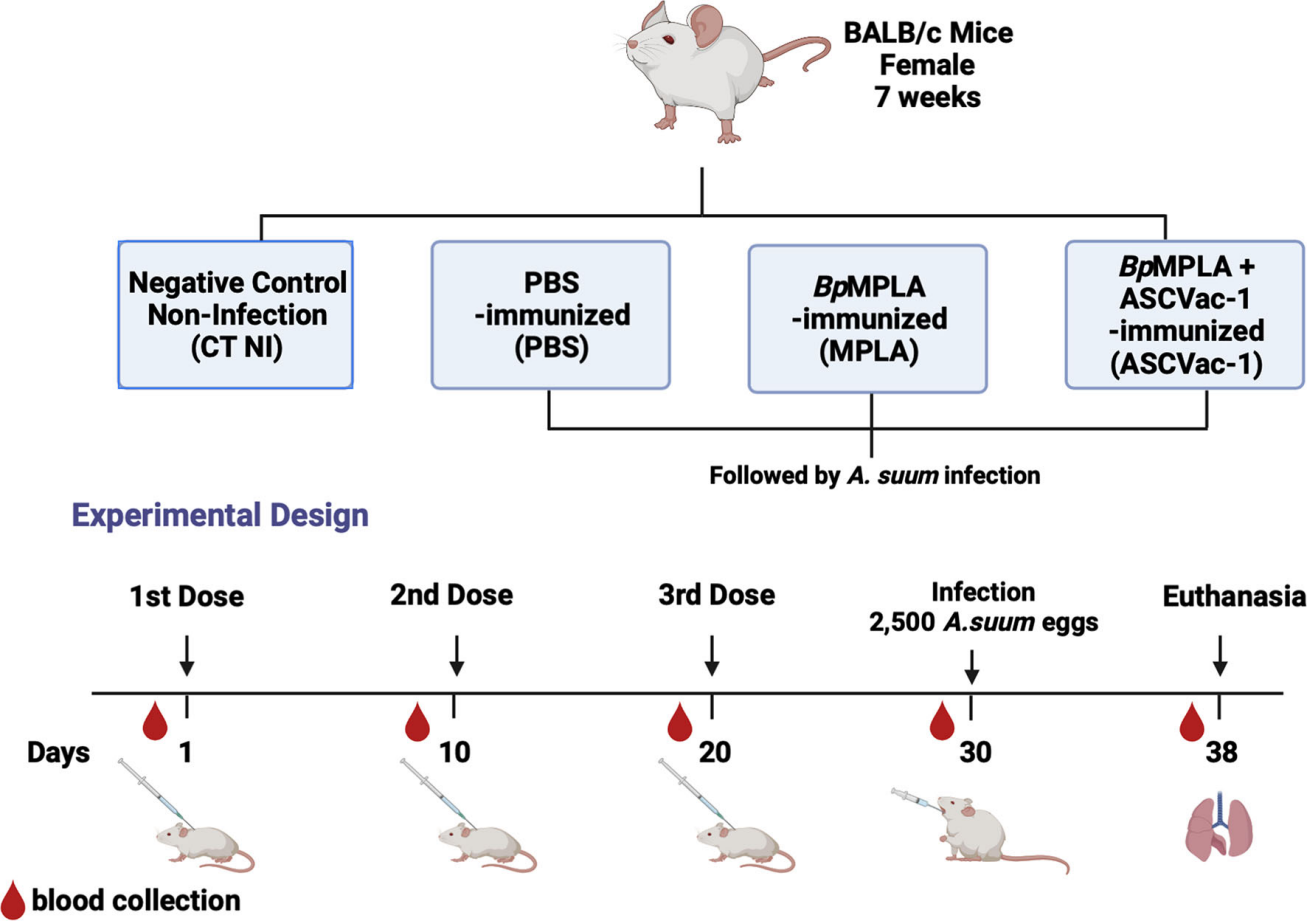
Experimental groups and experimental design of the *Ascaris* vaccination and infection scheme.

BALB/c mice (female, 7 weeks old) were obtained from the Central Animal Facility–Federal University of Minas Gerais, Brazil, and divided into four experimental groups with 16 mice per group. The first group was the control group without immunization and infection (CT NI). The mice in the second group received PBS only (PBS). Following our immunization protocol described in the previous study (16), the mice in the third group each received 25 μg of the adjuvant *Bp*MPLA (Monofosforil lipid A from *Bordetella pertussis*, Butantan Institute) as adjuvant control (MPLA). The mice in the 4^th^ group were each immunized subcutaneously with 25 μg of the chimera ASCVac-1 formulated with 25 μg *Bp*MPLA (ASCVac-1+MPLA) in a total volume of 130 µl. The mice were boosted twice with the same amount of antigen or adjuvant at 10 days intervals. Ten days after the last immunization, mice in groups 2, 3, and 4 were challenged with 2,500 *Ascaris* fully embryonated infective eggs in a total volume of 0.2 ml by oral gavage. Just before challenge, the fully embryonated eggs of *A. suum* were treated with 5% (v/v) sodium hypochlorite in an incubator (37°C and 5% CO_2_) for 2 h to disrupt the outer layer to facilitate *in vivo* larval hatching. After the incubation, the eggs were washed in PBS five times and counted under a microscope. Blood was collected from each mouse before each immunization and challenge, and serum was isolated for antibody measurement. The immunization regime is described in **Figure 2**.

### ELISA for Detection of Specific IgG, IgG Subtypes, and IgE Production

ELISAs were performed to measure the vaccine-specific antibodies in the sera of mice after each immunization with the chimera ASCVac-1 and the controls (CT NI, PBS, and MPLA). Briefly, ELISA plates (Greiner-Bio-One, USA) were coated with 1 µg of purified chimera protein ASCVac-1 in a total volume of 100 µl per well overnight at 4°C. On the following day, plates were washed five times with the washing buffer (PBS-0,05% Tween20) and then blocked with 250 μl of PBS containing 3% BSA for 1 h at 37°C. The antigen-coated plates were incubated with 100 μl of the sera of mice from each group diluted at 1:1,000 in PBS-BSA 3% per well for 2 h at room temperature. After being washed five times with washing solution, 100 μl of HRP conjugated anti-mouse IgG diluted at 1:2,000 in PBS-BSA 3% was added into each well. After 1 h of incubation at 37°C, the plates were washed and developed with 100 μl of the developing solution containing 0.1 M citric acid, 0.2 M Na_2_PO, 0.05% OPD (o-phenylenediamine dihydrochloride), and 0.1% H_2_O_2_. The plates were incubated at 37°C for 20 min, and the reaction was stopped by the addition of 50 μl 0.2 M sulfuric acid. The resulting absorbance was read on an ELISA reader (VersaMax ELISA Microplate Reader/Molecular Devices, USA) using Softmax Pro 5.3 software at 492 nm. All assays were performed in duplicates.

For measuring IgG subclasses, the same ELISA protocol was used except for using HPR conjugated anti-mouse IgG1, IgG2a, and IgG3 (Southern Biotech, USA) diluted at 1:1,000 in PBS-BSA 3%. Sera from the vaccinated groups were diluted at 1:500 for IgG1, IgG3, and 1:100 for IgG2a. To determine IgE levels, serum samples were diluted 1:10 and the HRP-conjugated anti-mouse IgE (Southern Biotech, USA) was diluted 1:500 in PBS-BSA (3%).

### Vaccine Efficacy: Parasitological Analysis

Eight days post-challenge with 2,500 *A. suum* infective eggs, each mouse in Groups 2, 3, and 4 was euthanized and lung tissue was collected. The tissue was minced into small pieces with surgical scissors, and then placed in a modified Baermann apparatus and incubated in PBS at 37°C and 5% CO_2_ for 4 h. The larvae precipitating to the bottom of the apparatus were collected and fixed in 4% formalin. The number of larvae from each mouse lung was counted under a microscope, and larval reduction in the vaccinated group was calculated compared to the adjuvant control group. To collect *Ascaris* larvae in the bronchoalveolar compartment of infected mice, eight animals from each group were anesthetized. BAL fluid was collected from each mouse by inserting a 1.7 mm catheter into the trachea and washing it with 1 ml of PBS two times. The collected BAL fluid was filtered using a cell strainer of 40 µm to purify *A. suum* larvae, and the remaining flowthrough fluid was centrifuged at 3,000 × g for 10 min. The larvae in the pellet were counted using a microscope, and BAL fluid supernatants were used to quantify total proteins, hemoglobin, and inflammatory mediators.

### Measurement of Exuded Proteins and Hemoglobin in Bronchoalveolar Lavage Fluid

The concentration of total proteins present in BAL was determined by the bicinchoninic acid (BCA) method using a commercial kit (Pierce, USA) following the manufacturer’s instructions. A standard curve was performed using BSA, and the optical density (O.D.) value of the samples was aligned with the standard curve to determine the protein concentration. The results were expressed as μg of total protein per ml of BAL.

The hemoglobin concentration present in BAL fluid was measured by a colorimetric assay using the cyanometahemoglobin method. The reading was performed in a spectrophotometer at a wavelength of 540 nm using a modified Drabkin solution (Bioclin Quibasa, Brazil). The hemoglobin reagent (Bioclin Quibasa, Brazil) was used to prepare a standard curve in which the O.D. values of the samples were aligned to determine their concentration. Hemoglobin content was expressed in g of Hb per dl of BAL.

### Tissue Cytokine Profile and Quantification of the Activity of Eosinophils, Neutrophils, and Macrophages

To determine the tissue cytokine profile, the right lobe of the lung was removed from eight mice from each group, and 100 mg of tissue was homogenized by the Power-Gen 125 tissue homogenizer (Fisher Scientific, USA) in 1 ml of PBS supplemented with protease inhibitors (0.1 mM of methyl sulfonyl phenyl fluoride; 0.1 mM benzethonium chloride; 10 mM EDTA and 20 KI of aprotinin A) and 0.05% of Tween20. The homogenate was centrifuged at 800 × g for 10 min at 4°C, and the supernatant was used to determine the cytokines IL-4, IL-5, IL-6, IL-10, IL-13, and IL-33 by ELISA (R&D, USA) according to the manufacturer’s instruction. The absorbance of the samples was determined by VersaMax ELISA microplates (Molecular Devices, USA) at a wavelength of 492 nm.

The n-acetylglucosaminidase activity of macrophages (NAG), neutrophil myeloperoxidase (MPO), and eosinophil peroxidase (EPO) was evaluated in the homogenates of the lungs. After being homogenized using the Power-Gen 125 tissue homogenizer (Fisher Scientific, USA), the lung homogenate was centrifuged at 1,500 × g for 10 min at 4°C, and the resulting sediment was examined to determine the activity of EPO, MPO, and NAG.

### Histopathological Analysis

The left lobes of the lungs were removed from the eight mice of each group. The lungs were fixed in 4% formalin solution and gradually dehydrated in ethanol, then embedded in paraffin. The paraffin blocks were cut at 4–5 microns thick and stained with hematoxylin and eosin for evaluating tissue damage.

For the histopathological analysis in the lungs of animals after the migration of larvae through the organ, three analysis tools were used. The histopathological description in order to evaluate the lesions caused by larval migration in the pulmonary parenchyma of mice, and for that the infiltration of inflammatory cells and exudative phenomena were observed. In addition, semiquantitative and quantitative analysis was used to assess degree of airway inflammation, peribronchial inflammation, and presence of hemorrhage of the lungs. The difference between these two analyzes is based on the characterization of inflammation through an inflammation score previously described in the cited work, and the quantitative analysis is only to corroborate the semi-quantitative result, but this time analyzing the exact areas of each inflammation and its magnitude (16).

For quantitative analysis of inflammation in larva migrated lungs, peribronchial or perivascular inflammatory cells were captured and counted on a Motic 2.0 microcamera. The areas of peribronchial or perivascular inflammation were delimited with the aid of a cursor, segmented, processed, and the values of the respective areas in µm^2^ by using the Image J plus program.

### Assessment of Respiratory Mechanics

Pulmonary dysfunction was measured as previously described (19). Mice were anesthetized by subcutaneous injection of ketamine and xylazine (8.5 mg/kg xylazine and 130 mg/kg ketamine), tracheostomized, and connected to a computer-controlled ventilator (Forced Pulmonary Maneuver System R, Buxco Research Systems, Wilmington, NC, USA). Mice were ventilated at a rate of 160 breaths per minute under anesthesia. Under mechanical respiration, the Lung Resistance (Rl) was determined by Resistance and Compliance (RC) test. A fast-flow volume maneuver was performed, and the lungs were first inflated to +30 cm H_2_O and immediately afterward submitted to a high negative pressure to force the expiration until −30 cm H_2_O. The Flow-Volume curves were recorded during this maneuver. Suboptimal maneuvers were discarded, and for each test in every single mouse, at least three acceptable maneuvers were conducted to obtain a reliable mean for all numeric parameters.

### Statistical Analysis

Statistical analyses of the data were performed using the GraphPad Prism 7 software (GraphPad Inc, USA). To verify the distribution of the data, the Kolmogorov-Smirnov and Shapiro-Wilk tests were used. For analysis between two groups, the Student Test T was used for parametric data and Mann-Whitney U for non-parametric data. For analysis of variances between three or more groups, with only one qualitative variable, the One-way ANOVA tests followed by the Tukey and Bonferroni post-test (parametric data), or the Kruskal Wallis tests followed by Dunn’s post-test (non-parametric data). For analysis of variances between three or more groups, with two or more qualitative variables, the Two-way ANOVA tests followed by Tukey’s post-test were used. The test was considered significant when the p-value was ≤0.05.

### Ethics Statement

The maintenance and use of animals were carried out following the recommendations of the Brazilian College of Animal Experimentation (COBEA). The present study was approved by the Ethics Committee for Animal Experimentation (CEUA) of the Federal University of Minas Gerais, Brazil, through protocol #61/2018. All efforts were made to minimize animal suffering.

## Results

### Identification, Selection, and Validation of B-Cell Epitopes as Potential Vaccine Candidates

**Figure 3:**
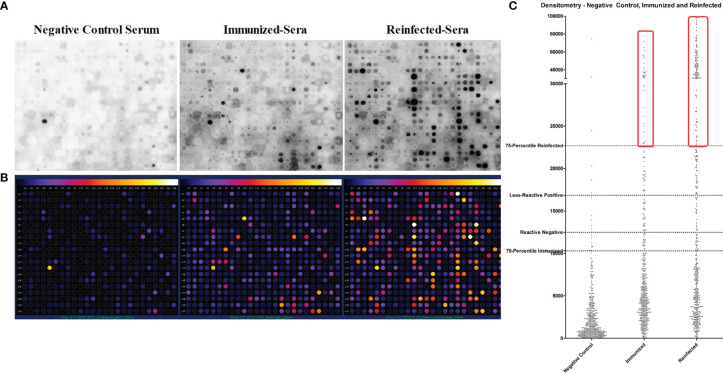
Helminth conserved B-cell epitope peptides recognized by *Ascaris*-immune sera by dot blot. **(A)** B-cell epitopes were synthesized as peptides and dotted on a membrane by Spot-Synthesis technique, the peptide dots were recognized by sera from BALB/c negative control mice (Negative Control Serum), from mice immunized with crude extract antigens of *A suum* (Immunized-Sera), and from mice infected with *A suum* infective eggs for three times (Reinfected-Sera). **(B)** Densitometry analysis of peptide spots recognized by sera of mice exposed to *A suum*. **(C)** Dispersion plot that represents the process of selection of highly specific peptides reactive to *Ascaris* antisera.

*In silico* prediction for helminth B-cell epitopes using a bioinformatic pipeline in 17-helminth proteome identified 480 conserved epitopes. These peptides were synthesized by the “Spot-synthesis” method and dotted on a cellulose membrane (**Figure 3A**). The peptide dots were probed with pooled sera from mice immunized with *A. suum* adult crude antigens (Immunized-Sera) (16), or mice infected with *A. suum* infective eggs three times (Reinfected-Sera), as well as the serum from normal control mice (Negative Control Serum). The peptide spots were scanned using densitometry to evaluate the signal as weak, medium, or strong after incubating with the *Ascaris*-immune sera in comparison with the negative sera. The intensity was converted to different colors by Image J- Protein Array Analyzer software allowing the characterization of the reactivity intensity of the peptides recognized by different immune sera compared to the normal mouse sera (**Figure 3B**). Those peptides below the cutoff value (background), or weak positive peptides (< cutoff + 3 SD) were excluded. Only those with intensity value ≥ cutoff + 3SD, without recognition by normal mouse serum, were selected as potential *Ascaris*-specific vaccine candidate peptides (**Figure 3C**). A total of 157 *Ascaris*-reactive peptides met the selection criteria. From these, the top 35 peptides with the highest reaction to *Ascaris*-immune sera with recognition intensity 20 times greater than the control sera were finally selected to be linked together by a KK linker in a sequence arrangement with optimal conformation and solubility to construct a multiepitope-based chimera vaccine antigen against *Ascaris* infection named ASCVac-1 (patent number: BR 10 2020 026968 2).

**Figure 4:**
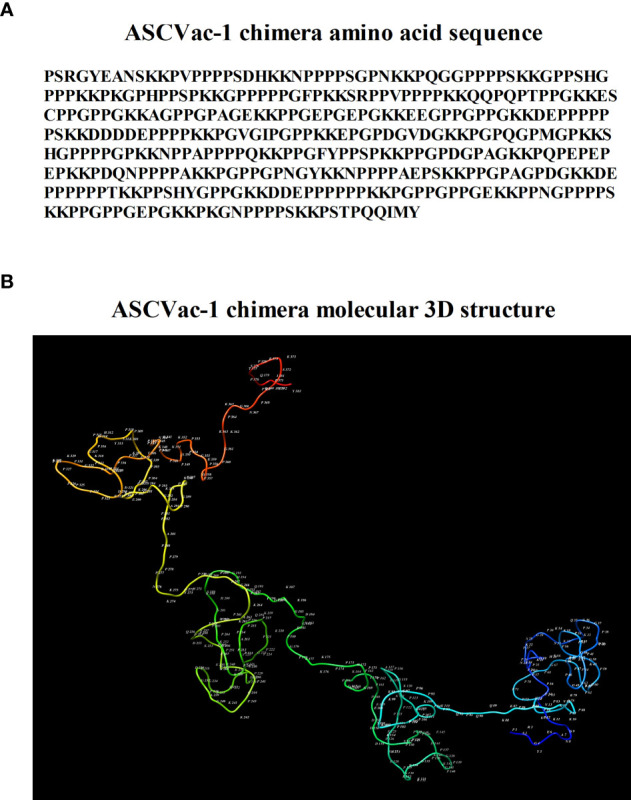
**(A)** Amino acid sequence of ASCVac-1, a multiepitope chimera vaccine candidate against *A suum* infection. **(B)** The 3D structure of ASCVac-1 was predicted by I-TASSER. Each letter and number represented in the structure presents the acronym of amino acid and its sequential order.

The sequence of ASCVac-1 is shown in **Figure 4A**. The 3D conformational structure of ASCVac-1 was predicted by I-TASSER. The model with the highest prediction score was selected and presented in **Figure 4B**.

### Chimeric Protein Construction, Expression, Purification, and Antigenicity

**Figure 5:**
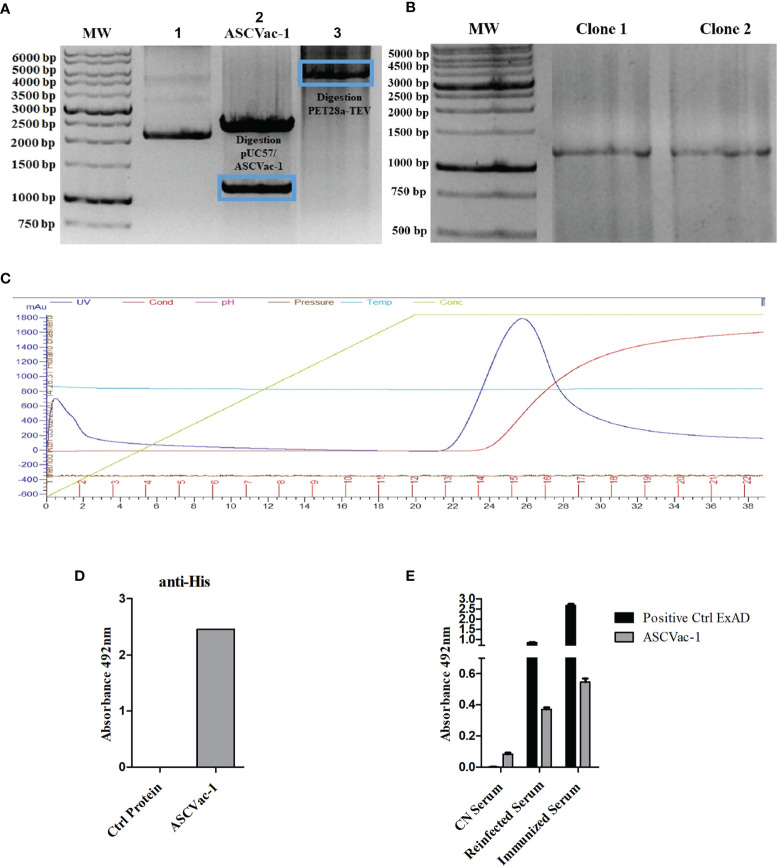
Cloning, expression, purification, and antigenicity assay of recombinant ASCVac-1 protein. **(A)** Electrophoretic agarose gel (0.7%) of the digestion product of plasmid. Lane 1, undigested pUC57+ASCVac-1; Lane 2, pUC57+ASCVac-1 plasmid digested with NdeI and HindIII releasing an insert of 1164 bp (blue frame); Lane 3, Expression vector pET28a-TEV digested with NdeI and HindIII. **(B)** 1% agarose gel of PCR products of ASCVac-1 insert in pET28a-TEV/ASCVac-1 plasmid extracted from transformed BL21 clone#1 and Clone#2. **(C)** ASCVac-1 protein affinity chromatographic purification. The peak absorbance for ASCVac-1 occurred between fractions 13 and 18. **(D)** The purified recombinant ASCVac-1 protein was highly recognized by anti-His antibody measured by ELISA. **(E)** Reactivity of ASCVac-1 chimera recombinant protein against sera of BALB/c mice negative control (CN Serum), or sera from mice with multiple exposures to infections by *A suum* (Reinfected Serum), and sera from mice immunized with crude extract antigens of *A suum* (Immunized Serum). The *A suum* crude antigen (ExAD) was used as positive control recognized strongly by both *Ascaris* immunized or infected sera.

The DNA encoding the chimeric protein ASCVac-1 was codon-optimized based on *E. coli* codon preference and synthesized by GenScript. The synthesized DNA was subcloned into the bacterial expression vector pET28a-TEV using NdeI and HindIII restriction sites. In the first lane of the gel, the plasmid pUC57+ASCVac-1 is found in the form of a closed plasmid without digestion by the enzymes. In the second lane, the plasmid pUC57/ASCVac-1 is digested with NdeI and HindIII, releasing the 1,164 bp ASCVac-1 insert (blue frame), and the 2,710 bp pUC57 vector. In lane 3, the expression vector pET28a-TEV was likewise digested with NdeI and HindIII (**Figure 5A**). After ligation of the insert and the linearized vector, the recombinant plasmid was transformed into *E. coli* BL21, and the transformants with the correct insert were confirmed by PCR (**Figure 5B**), and one of the positive colonies was used for large-scale expression. The expressed recombinant protein with a His-Tag at N-terminus was purified by affinity chromatography using the AKTA System (**Figure 5C**); it was successfully expressed as a soluble recombinant protein. The purified recombinant ASCVac-1 protein was recognized by the anti-His antibodies (**Figure 5D**).

The antigenicity of ASCVac-1 chimera against both *Ascaris*-immune sera (re-infected or crude Ag-immunized) and negative control (CN) sera were assessed by ELISA (**Figure 5E**). *Ascaris* crude antigens (ExAD) were used as a positive control of the assay. The ELISA results revealed that the chimeric ASCVac-1 protein was strongly recognized by both immune sera from mice immunized with *Ascaris* crude antigen (Immunized Serum) and infected with *A. suum* infective eggs (Reinfected Serum), but not by the sera from negative control mice (CN Serum). The ExAD has higher recognition better though by both mouse immune sera than the ASCVac-1 antigen.

### Immunization With ASCVac-1 Induced Specific Antibody Responses in Mice

**Figure 6:**
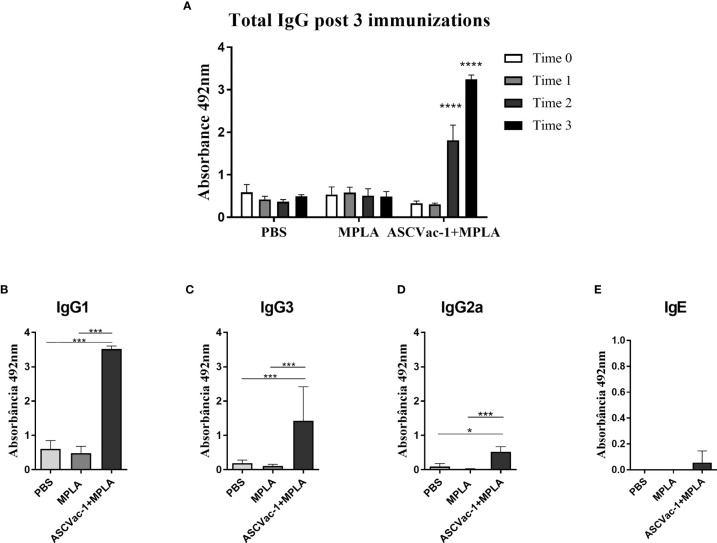
Antibody responses induced by the immunization with rASCVac-1. rASCVac-1-specific antibodies were measured by ELISA in BALB/c mice immunized with ASCVac-1. **(A)** ASCVac-1-specific IgG before first immunization (Time 0), post first immunization (Time 1), second immunization (Time 2), and third immunization (Time 3). **** were added to represent the statistical difference between antibody production after the 2nd and 3rd immunization with the ASCVac-1 chimera when compared to the control times. The ASCVac-1-specific IgG1 **(B)**, IgG3 **(C)**, IgG2a **(D)**, and IgE **(E)** in the sera of animals after the third immunization (Time 3) with chimera ASCVac-1+MPLA. Statistically significant differences *p<0.05, ***p<0.001 compared to PBS or MPLA groups.

To determine the immunogenicity of recombinant ASCVac-1 multiepitope chimeric protein, mice were subcutaneously immunized with 25 µg of rASCVac-1 formulated with 25 µg BpMPLA three times. Sera were collected from each mouse 10 days after the final immunization. The ASCVac-1-specific IgG level in sera started to increase after the 2^nd^ immunization and reached a high level after the 3^rd^ immunization. No anti-ASCVac-1 IgG antibody was detected in the sera of mice receiving PBS or MPLA adjuvant only (**Figure 6A**). The antigen-specific IgG subclasses and IgE levels were detected in the sera of each group after the 3^rd^ immunization. The ASCVac-1 immunization induced a marked IgG1 dominant response in the group of ASCVac-1+MPLA (**Figure 6B**). The IgG3 response was also significantly induced (**Figure 6C**), but with a relatively low IgG2a response (**Figure 6D**) in this immunization group compared with groups receiving PBS or MPLA. Results suggest that the ASCVac-1 antigen induces Th-2 polarized immune responses. Interestingly, immunization with ASCVac-1 three times did not induce antigen-specific IgE response (**Figure 6E**).

### ASCVac-1 Reduced the Lung Larva Burden in Mice Infected With *A. suum*

**Figure 7:**
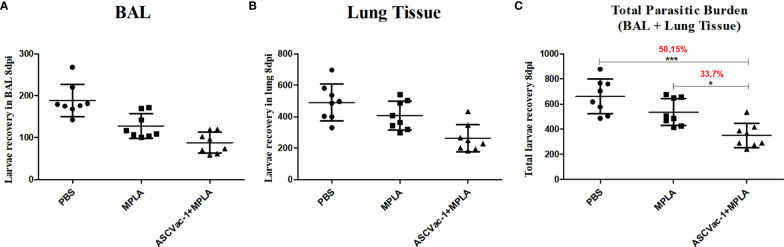
Immunization with chimeric ASCVac-1 formulated with MPLA reduced the larva burden in infected BALB/c mice. Larvae in BAL fluid and lung tissue were collected 8 days post the last immunization. **(A)** The number of larvae recovered from BAL fluid. **(B)** The number of larvae recovered from lung tissue. **(C)** The total number of larvae collected from BAL and lung tissue. The percentage value presented in the graph indicates the rate of reduction of the parasitic burden in relation to the control group PBS and the MPLA group. *p<0.05, ***p<0.001.

To investigate the preclinical efficacy of the chimeric ASCVac-1 vaccine and its capacity to protect against or reduce *Ascaris* infection burden, we counted the larvae collected from lungs and bronchoalveolar lavage fluid (BAL) of mice immunized with ASCVac-1+MPLA at day 8 of infection (peak of larval migration in lungs). Notably, ASCVac-1+MPLA-vaccinated mice showed a significant reduction in the larva burden in both BAL fluid (**Figure 7A**) and lung tissue (**Figure 7B**) when compared to PBS or adjuvant controls. Combining the number of larvae collected from BAL fluid and lung tissue, mice immunized with ASCVac-1+MPLA produced a significant larval reduction of 50.15% compared to the PBS control group (*p*<0.01) or 33.7% reduction compared to the adjuvant MPLA control group (*p*<0.05) (**Figure 7C**).

### Immunization With the Chimeric ASCVac-1 Protein Reduced Lung Hemorrhage and Inflammatory Cell Influx in the Airway

After observing a marked reduction of the parasite burden in animals vaccinated with the chimeric ASCVac-1, the next aim was to identify the potential mechanisms involved in this protection. Different arms of the immune response were characterized after immunization followed by infection, including the leukocyte influx profile into the airways, cytokine production, lung tissue histopathology, and lung mechanical function.

**Figure 8:**
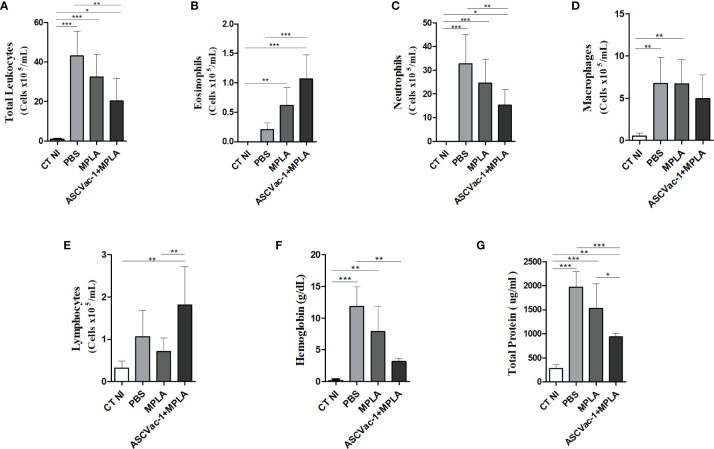
Vaccination with the ASCVac-1 chimera reduced inflammatory cell influx into the airways following *A suum* infection associated with increased lymphocytes and eosinophils. Total leukocytes and their classification in the bronchoalveolar lavage (BAL) in each group were quantified using a Neubauer chamber and cytospin preparations. **(A)** Total leukocytes. **(B)** Eosinophils. **(C)** Neutrophils. **(D)** Macrophages. **(E)** Lymphocytes. **(F)** Hemoglobin. **(G)** Total proteins. Statistically significant differences are represented by * compared to the negative control group CT NI, the infected control group PBS, and the MPLA group in which *p<0.05, **p<0.01, ***p<0.001.

To characterize the leukocyte influx into the airways, as well as the presence of hemoglobin (a marker for hemorrhage) and total proteins, the BAL fluid was collected from mice in different groups at day 8 of infection. Overall, during the infection, *Ascaris* larval migration not only caused lung damage and serious hemorrhage (increased hemoglobin and total protein in BAL fluid, **Figures 8F, G**) but also drove an intense influx of inflammatory cells to the lung tissue and airways, characterized by a marked increase of leukocyte numbers in the BAL (**Figure 8A**). However, mice immunized with ASCVac-1 significantly reduced the larva migration-caused lung hemorrhage (**Figures 8F, G**), associated with a significant reduction in the presence of these leukocytes in the BAL when compared to the infected PBS control group (20, 31 ± 11, 37 cells × 10^5^ *vs*. 43, 02 ± 12, 48 cells × 10^5^, p<0.01). After being classified, we found that the reduced leukocytes in the ASCVac-1 group were mostly inflammatory cells such as neutrophils (p<0.001) (**Figure 8C**) and macrophages (p<0.01) (**Figure 8D**) compared to the non-vaccine control groups. However, lymphocytes and eosinophils were significantly increased in the BAL fluid of mice immunized with ASCVac-1 compared to mice in PBS or MPLA control groups (p<0.01) (**Figures 8B, E**). Taken together, vaccination with ASCVac-1 not only reduced larval burden in the lungs but also reduced lung damage and inflammation of immunized mice. Increased eosinophils and lymphocytes may be related to protective immunity.

**Figure 9:**
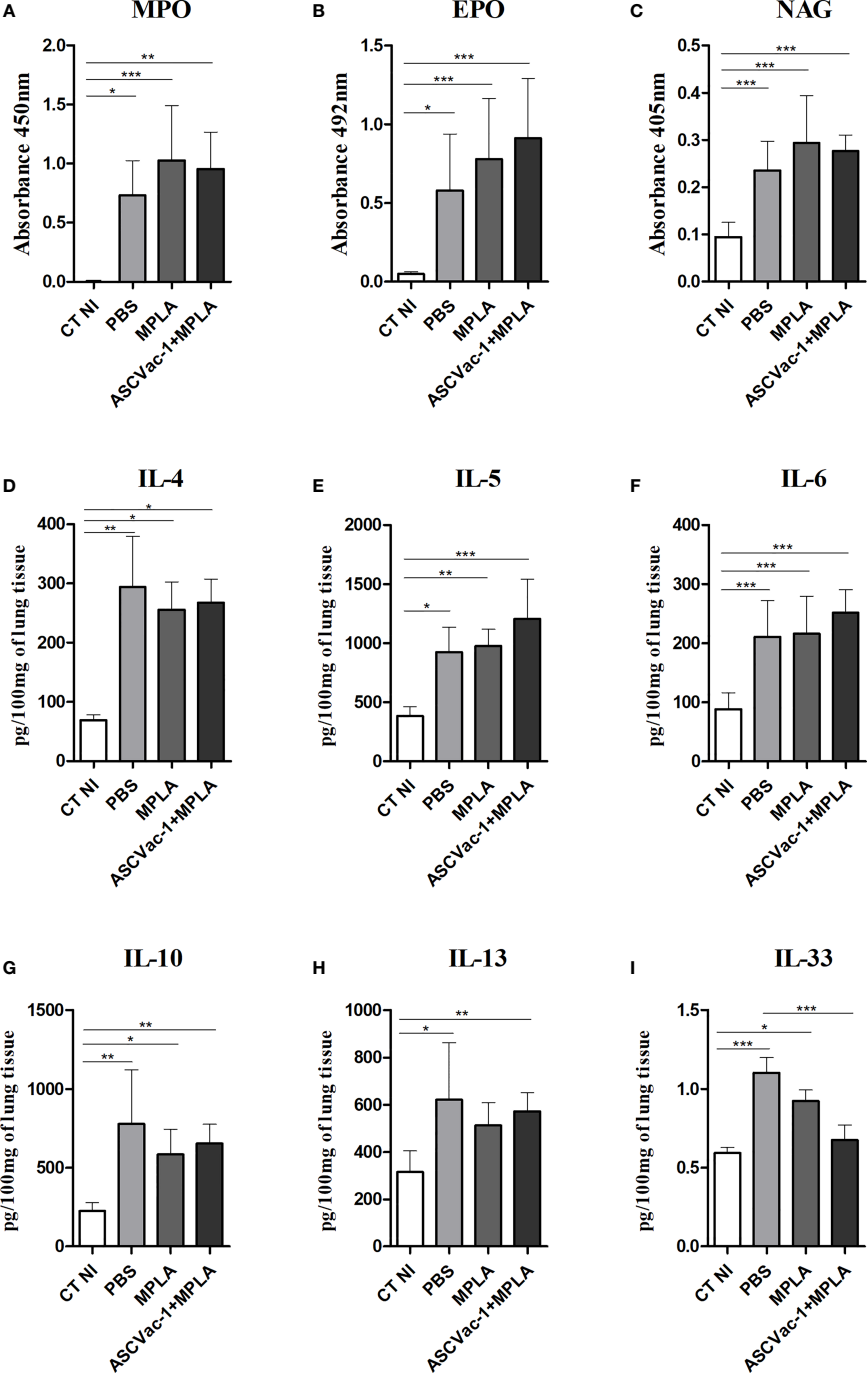
Pulmonary cytokine levels and cellular activity induced by vaccination with ASCVac-1 chimera following *A suum* infection. The levels of cytokines were quantified by ELISA. **(A)** MPO. **(B)** EPO. **(C)** NAG. **(D)** IL-4. **(E)** IL-5. **(F)** IL-6. **(G)** IL-10. **(H)** IL-13. **(I)** IL-33. Statistically significant differences (p<0.05) are represented by * compared to the negative control group CT NI and the infected control group PBS in which *p<0.05, **p<0.01, ***p<0.001.

No major differences were found in the levels of neutrophil myeloperoxidase (MPO) (**Figure 9A**), eosinophil peroxidase (EPO) (**Figure 9B**), and macrophage N-acetylglucosaminidase (NAG) (**Figure 9C**) when we compared the vaccinated with the unvaccinated mice upon *Ascaris* infection. Marked differences were observed in the groups infected with *Ascaris* when compared to the non-infected control group (CT NI).

To help characterize the immune response in the lung tissue and to compare the pulmonary cytokine signatures of ASCVac-1+MPLA-immunized mice followed by infection with PBS- or adjuvant-immunized mice followed by infection, we quantified the cytokines IL-4, IL-5, IL-6, IL-10, IL-13, and IL-33 in the lung homogenate. Overall, *Ascaris* infection in all groups induced a marked type-2 dominated immune response, with a significant increase in all cytokines assessed when compared with non-infected mice (CT NI) (**Figures 9D–I**). However, ASCVac-1+MPLA-immunized mice followed by *Ascaris* infection showed a marked reduction in the IL-33 levels in the lung tissue (similar to non-infected control mice) when compared with PBS-immunized mice followed by *Ascaris* (0, 6745 ± 0, 09743 pg/100 mg *vs*. 1, 102 ± 0, 09670 pg/100 mg, p<0.001). 

### Vaccination With ASCVac-1 Reduced Lung Damage and Induced Less Dysfunction of Pulmonary Mechanics

**Figure 10:**
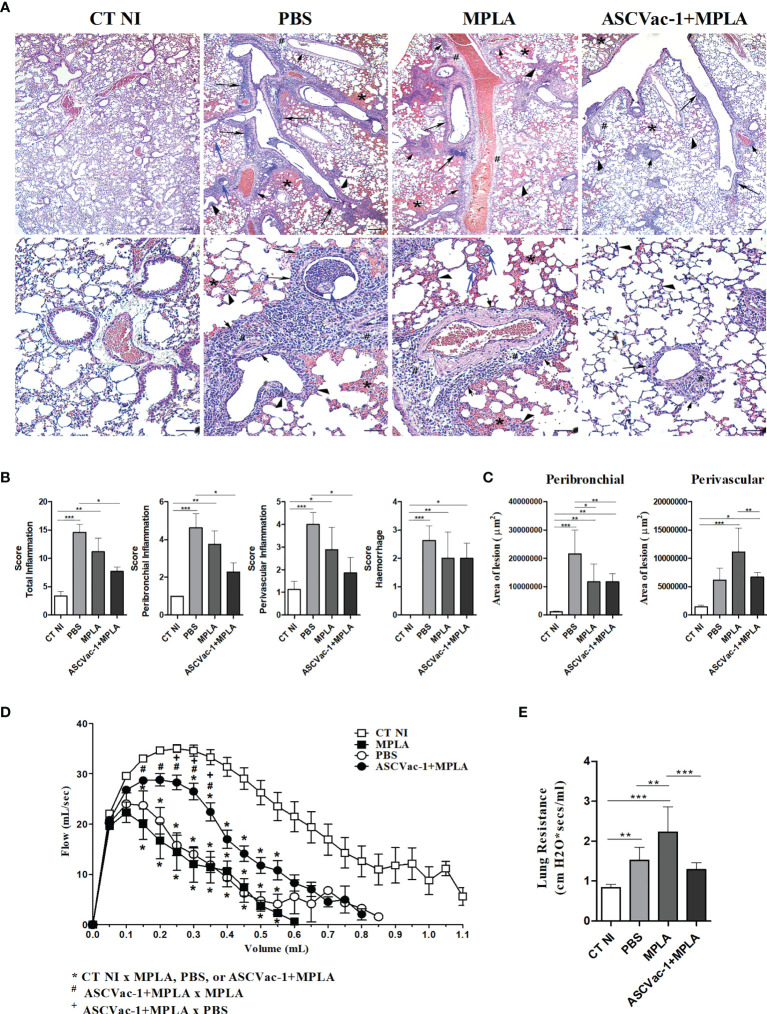
Improved histopathology and pulmonary mechanical function in mice vaccinated with ASCVac-1 chimera. **(A)** Histochemical staining of lung tissues collected from different group mice. The arrowheads indicate infection caused thickening of interalveolar septa; # indicates perivascular edema; short arrows indicate perivascular inflammatory infiltration; arrows indicate intense inflammatory infiltration around the lower airways; blue arrow indicates *Ascaris suum* pulmonary L3 larvae; * indicates the presence of exuberant hemorrhage. Bar = 500 μm, Bar = 200 μm. **(B)** Semiquantitative evaluation of pulmonary inflammation and hemorrhage in each group. **(C)** Quantitative measurement of pulmonary lesion area caused by *A suum* larva infection in different groups of mice. Pulmonary dysfunction was evaluated by analysis of **(D)** Flow-volume curve and **(E)** Lung resistance. *p<0.05, **p<0.01, ***p<0.001.

To evaluate the effect of ASCVac-1 vaccination on the reduction of pathology in the lung caused by the *A suum* larva infection, the histopathological analysis of the pulmonary parenchyma including topography, inflammatory infiltrate, presence or absence of larvae, and vascular and exudative phenomena was performed in the sections of lung tissues collected from different groups of mice (**Figure 10A**). After being infected with *A. suum* infective eggs for 8 days, lung tissues demonstrated significant exudative phenomena characterized by perivascular edema, hemorrhage, and mixed inflammatory cells infiltration around vessels, bronchi, and bronchioles in mice of PBS and MPLA groups. Hypertrophy and hyperplasia of the epithelium cells of the bronchi and bronchioles were frequently observed. The presence of pulmonary L3 larvae in or near hemorrhagic areas and the lumen of bronchi and bronchioles was also observed. However, in mice immunized with ASCVac-1+MPLA, the lung inflammation and damage were significantly reduced with less inflammatory cell infiltration, less exudative phenomena, and hemorrhage. The most infiltrated cells were lymphocytes and macrophages rather than eosinophils and neutrophils. In addition, few infected larvae in lung tissue were observed compared to non-immunized groups.

A semiquantitative analysis based on the pathological score identified that scores based on the total inflammation, peribronchial inflammation, and perivascular inflammation were significantly reduced in the group of mice immunized with ASCVac-1 + MPLA compared to PBS or MPLA groups (p<0.01), and the score of hemorrhage reduced only compared to PBS group (**Figure 10B**). The perivascular lesion area was also significantly reduced in the immunized group compared to the MPLA control group, but peribronchial lesion reduction was only observed between the immunized group and PBS control group (**Figure 10C**).

Dysfunction of pulmonary mechanics is used as a marker of tissue injury. Thus, we evaluated the impact of immunization with ASCVac-1 on the improvement of lung function compared to control mice. The analysis of the Flow-Volume curve revealed that airflow through airways was significantly reduced in mice infected with *A. suum* infective eggs (PBS or MPLA groups) (**Figure 10D**). Immunization with ASCVac-1 greatly improved the airflow of mice compared to mice receiving PBS or MPLA only (**Figure 10D**). The Lung Resistance evaluation also demonstrated that mice infected with *A. suum* infective eggs (PBS or MPLA) increased the airway resistance and reduced the elasticity of infected lungs, and immunization with ASCVac-1 significantly improved the lung elasticity affected by *Ascaris* larva infection (**Figure 10E**). The improved lung function results correlate with our findings of improved tissue pathology in ASCVac-1 immunized mice (**Figures 10A** **–C**). Collectively, mice immunized with ASCVac-1 experienced improved lung function by increasing airflow and reducing airway resistance correlating with reduced lung pathology.

## Discussion

The current strategy for human ascariasis control is predominantly based on mass drug administration (MDA) with a benzimidazole class of anthelmintics that are effective in removing active helminth infections. However, MDA does not provide long-lasting protection against *Ascaris* spp. infection, allowing high rates of re-infection shortly after treatment. Thus, individuals living in endemic areas are rapidly reinfected, even after successful treatment (20). In this way, the development of a vaccine that provides long-term protection against *Ascaris* or at least reduces the parasite burden to lower morbidity in the population living in endemic areas should be considered as the main control strategy for *Ascaris* infection.

The immunological basis for the development of an effective vaccine against human *Ascaris* sp. infection gained strength after studies using experimental infection in animal models were performed, which demonstrated that a protective immune response could be developed after repeated infection or following multiple exposures to the parasite. This protective immunity is associated with a marked reduction in parasite burden (72–90%), as well as reduced morbidity (21–23). The mechanism underlying the protective immunity against *Ascaris* sp. is linked with an eosinophil-dominated type 2 mucosal response (24, 25), combined with a systemic mixture of Th2/Th17 (21) and IgG *Ascaris*-specific antibodies (16). However, there is no vaccine available for *Ascaris* sp. infection yet that triggers such a protective response. Thus, the use of new technologies, such as the production of recombinant proteins, the construction of synthetic genes, and the evaluation of peptide-based chimeras, will accelerate the selection, characterization, and validation of potential vaccine candidates. For this study, a comprehensive cutting-edge strategy was used to select B-cell-targeted epitopes from 17 common intestinal helminths for the development of a pan-helminth vaccine, resulting in the selection of 35 highly reactive peptides specific to *Ascaris*. These peptides were linked together in a sequence arrangement with the best conformation to construct a polypeptide chimera called ASCVac-1 in this study. This chimera vaccine construct was expressed as a recombinant protein that was used for vaccination of BALB/c mice and subsequent challenge with *Ascaris*-infective eggs. The vaccine efficacy and immunological mechanism underlying the protection of ASCVac-1 were systematically evaluated in immunized mice based on the parasitological, physiological, and immunopathological characterization of the lung tissue and the systemic immunological responses (humoral and cellular).

The immunization of BALB/c mice with the ASCVac-1 chimera resulted in a significant reduction of the parasitic burden (50%) when compared to animals that received PBS. Even though several *A. suum* larval antigens such as As14 (7), As16 (8, 9), As24 (10), As37 (11, 12), enolase-1 (13), and AsPPase (14) expressed in different recombinant protein systems induced a certain degree of protection against *A. suum* infection in a mouse model, that protection was not complete and worm reduction was low. The combination of these protective antigens as a multivalent vaccine could be a strategy to enhance protective immunity. Based on this approach, de Castro et al. (15) recently constructed a chimeric antigen based on the B-cell epitopes from protective antigens As37, As16, and As14 (15). Notably, mice immunized with this new chimeric protein alone (without adjuvant) showed a 42.9% reduction in larval burden compared to the control group after challenge with *Ascaris* eggs (15). These data, together with the increased efficacy observed in our study, suggest that chimeric proteins containing more than one immunogenic epitope offer a new platform for the development of vaccines against *Ascaris* and other helminths. In both studies, the protective immune responses were associated with the development of a robust antibody response to control the parasitic burden, which is corresponding with the constructs made of B-cell epitopes.

The important role of the Th2 immune response, especially the parasite or antigen-specific antibodies, in the protection against helminth infections has been well studied and understood (15, 16, 26–29). We also found that ASCVac-1 vaccination induced high levels of IgG, with a significant and gradual increase of IgG1 and IgG3 subclasses after the second dose of the chimera immunization. This finding is consistent with studies from Khoury et al. (30), McCoy et al. (31), and Gazzinelli-Guimarães et al. (16), which have shown that prolonged exposure to helminth infections by infective eggs or multiple immunizations with *Ascaris* antigens induced the generation of specific IgG antibodies, with a predominance of IgG1 (16, 30, 31). Interestingly, similar to the results from Islam et al., 2005 (10), immunization of ASCVac-1 did not induce any detectable IgE response, suggesting that the protective immunity induced by the ASCVac-1 is not necessarily mediated by IgE, and more importantly, the immunization with ASCVac-1 seems to be safe for not eliciting IgE-sensitization and IgE-related allergic response in the immunized mice.

Regarding the cellular response driven by the ASCVac-1 chimera followed by *Ascaris* infection, we observed a significant increase in the numbers of lymphocytes as well as eosinophils in infected lung tissue. It has been described that the presence of helminth larvae in the lungs triggered rapid eosinophil recruitment and activation by the induction of elevated levels of IL-5 and Eotaxin-1 (CCL-11) (a chemoattractant for eosinophils) (32–35). Interestingly, Gazzinelli-Guimaraes et al. (25) demonstrated that house dust mite allergen induced high production of eosinophils that protected mice from *Ascaris* larval infection; however, this protection was not observed in eosinophil-deficient mice, indicating eosinophil-dominated type-2 immune response is associated with protective immunity against *Ascaris* infection. Another important result observed in our study was the downregulation of the IL-33 levels in the ASCVac-1 immunized mice followed by *Ascaris* infection. IL-33 is triggered by epithelium damage driven by the *Ascaris* larval infection during their migration through the lungs into the airways, and the level of IL-33 was correlated with the worm burden (12, 25). The reduced levels of IL-33 in the ASCVac-1 immunized mice were associated with reduced larva number in the lung, as well as attenuated inflammation, lung damage, and dysfunction, indicating IL-33 is a marker of protection against *Ascaris*.

Immunization with ASCVac-1 not only significantly reduced larva burden in the lung of mice challenged with *A. suum* infective eggs but also reduced lung damage and improved lung functions affected by infected *Ascaris* larvae. Mice immunized with ASCVac-1 significantly reduced hemoglobin and total proteins in the BAL fluid of the lung in comparison with PBS-immunized mice, suggesting less bleeding and lung tissue damage caused by the *Ascaris* larval migration (exudation and extravasation of blood and proteins) and decreased morbidity caused by the rupture of blood vessels during the larval migration. The larva migration in the lungs of infected mice causes lung tissue damage and pulmonary inflammation that harms the lung function of infected mice. After immunization with the ASCVac-1 chimera, mice demonstrated a significant reduction in tissue inflammation characterized by significantly reduced neutrophil inflammatory cell infiltration. Instead, the neutrophil filtration was converted to mononuclear cells, mainly with lymphocytes, macrophages, and eosinophils, indicating that these cells may contribute to limiting the parasite infection and recovery/healing of the damaged lung tissue. All these improved immunological and pathological changes in ASCVac-1 immunized mice may contribute to improved lung functions, including improved airflow, lung elasticity, and reduced airway resistance. These data further suggest that the vaccine played a fundamental role in the preservation of the organ, avoiding severe tissue damage that could lead to impaired pulmonary function of infected animals.

In this study, by using *in silico* prediction we identified 480 B-cell epitopes from 17 common helminths that infect humans and animals. This pipeline of B-cell epitopes led to the identification of 35 epitopes highly recognized by *Ascaris*-immune sera, resulting in the construction of the ASCVac-1 vaccine candidate that induced significant protection in this study. Since these 480 B-cell epitopes exist in all 17 helminths, it is a feasible and useful tool used to identify vaccine or diagnostic epitopes for any other helminth infection by screening with the corresponding infected or immune sera or even to identify pan-helminth vaccine candidate(s) that can be used to prevent co-infection of several helminths as common in endemic areas (36).

In our previous work, we demonstrated that the immunization with different crude antigens derived from *A. suum* were able to reduce by up to 60% of the parasitic burden after challenge. Parasite derived crude antigen-based vaccines are excellent models for developing proof of concepts for vaccines against parasites, and mostly, for understanding the protective mechanisms, and to characterize the targeted immune responses. However, working with crude antigens as potential vaccine targets has many disadvantages, including the lack of standardization, high variability on different lots, unspecific targets, and high risk for inducing a bystander non-desire response, such as IgE levels. All these disadvantages are possible to be overcome by using prediction based bioinformatic tools, but maintaining the vaccine efficacy around 50%, which is considerable as highly effective in controlling the parasite intensity, reducing the pulmonary inflammation, and promoting morbidity control. In addition, by using bioinformatics tools, it was possible to select highly immunogenic epitopes to *Ascaris*, with conserved sequences among different helminth parasites of public health importance. Thus, by using this tool, the development of a pan-vaccine against helminth parasites became more real and less expensive.

Taken together, the preclinical trial with the ASCVac-1 chimera in mice demonstrated its significant vaccine efficacy against *Ascaris* infection associated with strong IgG-based Th2 responses, but with no IgE induction, thus reducing the risk of allergic response in vaccinated subjects. All results suggest that the multiepitope-based ASCVac-1 chimera is a promising vaccine candidate against *Ascaris* sp. infection. The epitope-based reserve vaccinology approach constitutes an alternative technology to develop a multivalent pan-helminthic vaccine to support global STH elimination programs. A pan-helminthic vaccine would be a major step in the eradication of several parasites that share significant geographical overlaps and in reducing the morbidity of helminth infections in endemic regions (36).

## Data Availability Statement

The original contributions presented in the study are included in the article/supplementary material. Further inquiries can be directed to the corresponding author.

## Ethics Statement

The animal study was reviewed and approved by Comissão de Ética no Uso de Animais–CEUA #61-2018 (Universidade Federal de Minas Gerais).

## Author Contributions

Conceived and designed the experiments: AG-G, LB, RF. Performed the experiments: AG-G, DN, CA, FO, AC-D-S, SC, LK, VF, FS, JC. Analyzed the data: AG-G, DN, LB, RR, RF, MB, PJH, BZ. Contributed reagents/materials/analysis tools: AG-G, FB, LB, RF, DB, MA, PLH. Wrote and reviewed the paper: AG-G, LB, RF, RR, BZ, PLH. All authors contributed to the article and approved the submitted version.

## Funding

This work was financially supported by the Fundação de Amparo a Pesquisa do Estado de Minas Gerais/FAPEMIG, Brazil (Grant# CBB APQ-03280-15, Rede Mineira de Imunobiológicos RED-00140-16), the Brazilian National Research Council (CNPq) (Grant# 303345/2018-7 and Grant# 442994/2019-2), Pró Reitoria de Pesquisa of Universidade Federal de Minas Gerais and CAPES. AG-G was supported by a PhD fellowship from the CNPq. RF, LB, RR, and DB are CNPq Research Fellows (Bolsa de Produtividade em Pesquisa).

## Conflict of Interest

AG-G and RF are inventors in a patent related to the chimeric protein against *Ascaris* sp. infection (Brazilian INPI Protocol #BR10 20200269682).

The remaining authors declare that the research was conducted in the absence of any commercial or financial relationships that could be construed as a potential conflict of interest.

## Publisher’s Note

All claims expressed in this article are solely those of the authors and do not necessarily represent those of their affiliated organizations, or those of the publisher, the editors and the reviewers. Any product that may be evaluated in this article, or claim that may be made by its manufacturer, is not guaranteed or endorsed by the publisher.
